# Supplementary material for: Emotional profiling and cognitive networks unravel how mainstream and alternative press framed AstraZeneca, Pfizer and COVID-19 vaccination campaigns
Source: Sci Rep. 2022 Aug 24;12:14445. doi: 10.1038/s41598-022-18472-6 (PMC9400577; doi:10.1038/s41598-022-18472-6)
Supplement: Supplementary file 1 — Supplementary Information. [file 41598_2022_18472_MOESM1_ESM.pdf]

Supplementary material for “Emotional profiling and cognitive networks unravel how mainstream and alternative press framed AstraZeneca, Pfizer and COVID-19 vaccination campaigns”

Alfonso Semeraro[1], Salvatore Vilella[1], Giancarlo Ruffo[1], and Massimo Stella[2,*]

[1]University of Turin, Computer Science Department, Turin, 10149, Italy
[2]CogNosco Lab, Department of Computer Science, University of Exeter, Exeter, EX4 4QG, UK
[*]m.stella@exeter.ac.uk

1 On the prevalence of mainstream and alternative news on social networks

We first showcase some general, descriptive statistics about the prevalence and popularity of the discourse about vaccines on social media. As explained in Sec. Methods, when collecting articles we distinguished between *mainstream* and *alternative* media outlets, having set the objective of finding whether there are significant differences in the narrative around vaccines between the two categories.

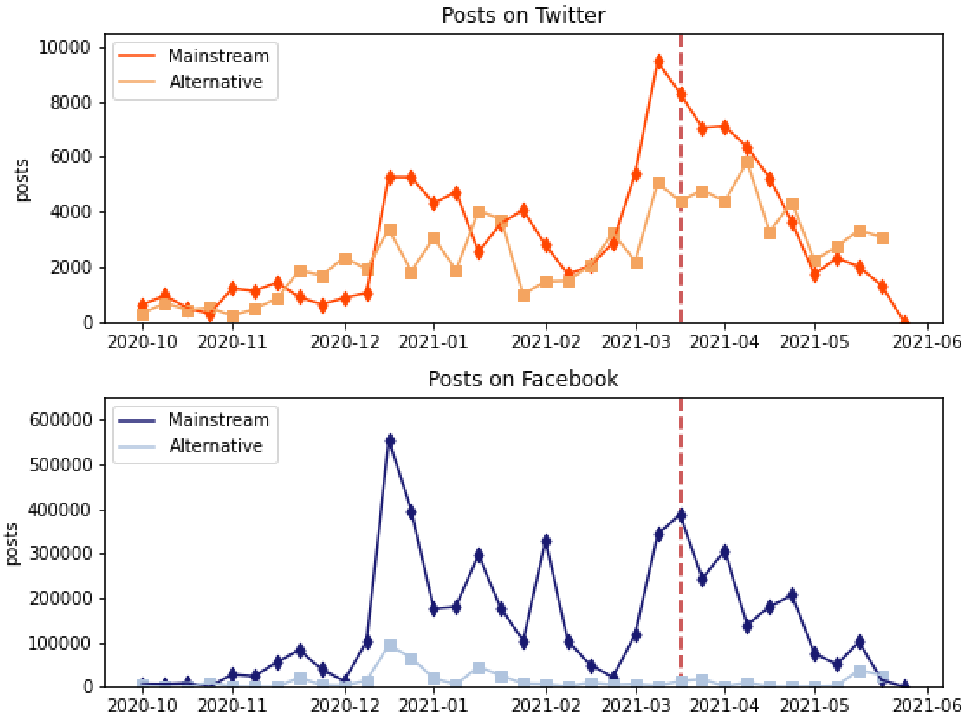

Figure 1. Posts on Twitter and Facebook in the dataset. A peak is registered in week 25 of observation, around the suspension of AstraZeneca on March 15.

Figure 1 reports the amount of shares on Twitter (top) and Facebook (bottom), divided by mainstream and alternative media sources, and grouped by week. The activity on the two social networks is substantially different in numbers, with an average of 153 thousand shares by week on Facebook and only 5 thousands on Twitter, but it shows some resemblances. The number of daily posts slowly rises in late 2020, when the first news about the trial of Pfizer and AstraZeneca appeared; it bumps at the end of December 2020, when first Pfizer was approved for subministration[1], and later at the end of January 2021, when AstraZeneca (now VaxZevria) was also approved[2] by AIFA, the Italian Agency for Drugs. The official press releases from

AIFA are responsible for outstanding peaks of activity on Facebook (bottom), exactly around the approval of both vaccines. The number of posts diminishes in February, but it quickly rises to the highest level on March, around the suspension of AstraZeneca of March 15 (red dotted vertical line) due to a small number of suspect cases of thrombosis in Europe[3]. While consistently higher than before, the volume of the discussion around both vaccines decreased steadily after March 15. While the general trends of engagement are similar on both social networks, there is a striking difference on what kind of sources drove the conversation about vaccines. The shares of news published by alternative sources can be considered negligible on Facebook, but on Twitter such news represent a massive quota of the total shares. To give a numeric comparison, total shares of alternative sources contents on Facebook were 8.36% of the total, while on Twitter 43.61%. Notice that Facebook counts might be underestimated because of sampling issues (see Methods) that originate from alternative sources being spread in smaller social pages that can go undetected by our external data gathering tools. On Twitter, where user activities are transparently monitored by the Twitter API (i.e., no third-party software) data exhibit no evident posting differences between alternative and mainstream news sources. The same high level of agreement is consistently present in mainstream news across Facebook and Twitter.

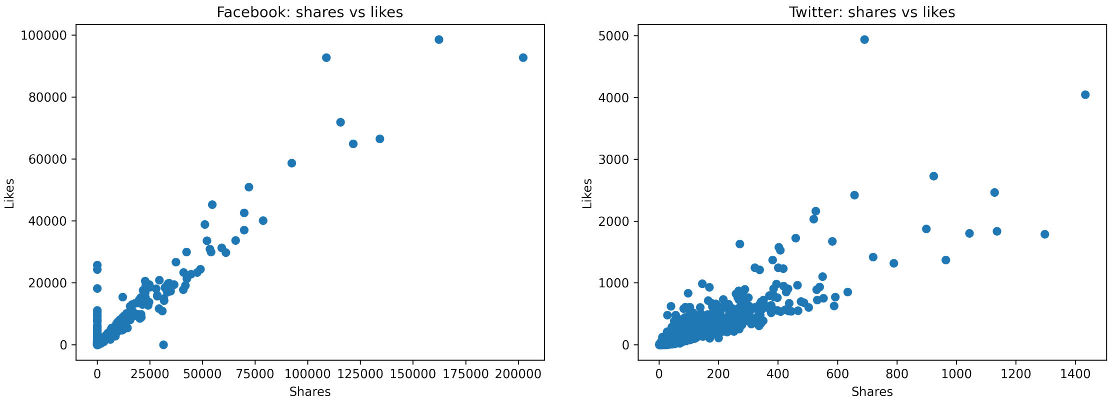

Figure 2. Correlation between number of likes and number of shares of a URL on Facebook (left) and Twitter (right). On both social networks, the two measures are strongly correlated (Pearson 0.96 and 0.88, p-value $\ll 0.001$.)

Fig. 2 shows the trend between the number of likes of a URL and the number of times it was shared on Facebook (left) and on Twitter (right). The two measures are strongly correlated (Pearson 0.96 and 0.88, p-values $\ll 0.001$). A Wilcoxon test confirmed that articles shared on Twitter have been liked more times than on Facebook, on average (see Section 3.1), and it holds for the overall case (sample size = 977, statistic = 628), as well as for the mainstream news (sample size = 857, statistic = 448) and for alternative news (sample size = 120, statistic = 12). In all cases $p \ll 0.001$. Please note that the coverage of ShareScore (cfr. Section 2) and the Facebook's graph APIs we used to retrieve such an information is partial, hence the reduced sample size.

2 On the characteristic distribution of emotions in the dataset

The distribution of emotions around the word AstraZeneca displayed in Fig. 1 (D) is peculiar, and it differs from the distribution of emotions found in general, non covid-related news. We collected a sample of 3400 articles coming from the same venues, but on different topics (we discarded those that contained keywords such as "covid", "vaccine", "astrazeneca", "pfizer"...). We analysed both titles and bodies of news coming from mainstream and alternative sources. Results are displayed in the Figures 3 and 4.

Mainstream bodies exhibit high levels of anticipation and trust, but also of joy and surprise, which was not present in Fig. 1 (D), and less disgust than the baseline. Alternative news bodies show anticipation and trust as well. Neither of them shows the low level of anger we observed in the AstraZeneca's neighborhood.

Mainstream titles resemble mainstream bodies, but without the prominent surprise and with less sadness than the baseline; alternative titles are still dominated by anticipation and trust, but also surprise, and they show low disgust.

From Figures 3 and 4 appears that the distribution of emotions surrounding AstraZeneca is peculiar. Furthermore, the similarity between mainstream and alternative bodies does not seem to hold for the general news system.

The distribution of emotions around AstraZeneca is also dependent on the partition of titles in mainstream and alternative, rather than on the underlying distribution of emotions in our dataset. We operated 50 random partitions of the titles in two lists,

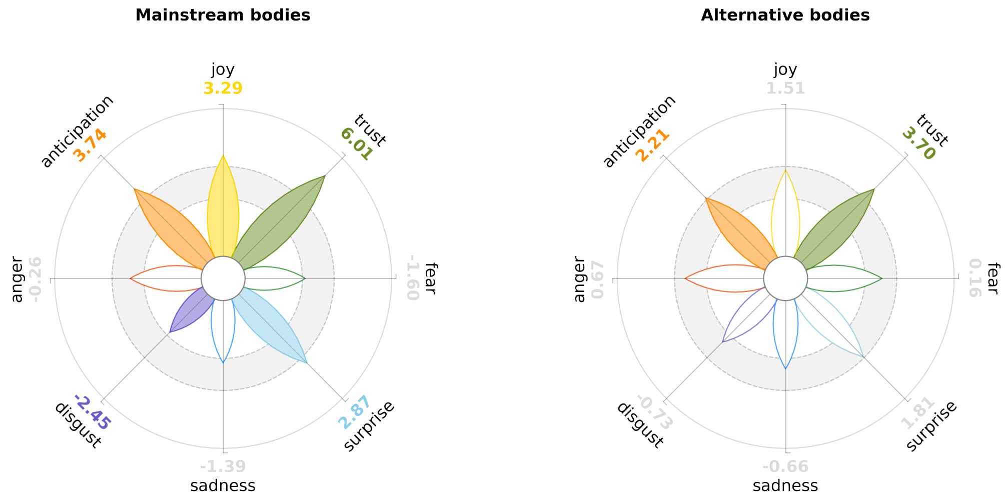

Figure 3. Emotions in mainstream and alternative bodies. Along with anticipation and trust, non-covid mainstream news exhibit high surprise and low disgust. anticipation and trust are a trait of alternative news' bodies as well. These distributions significantly differ from those in Fig. 1 (D).

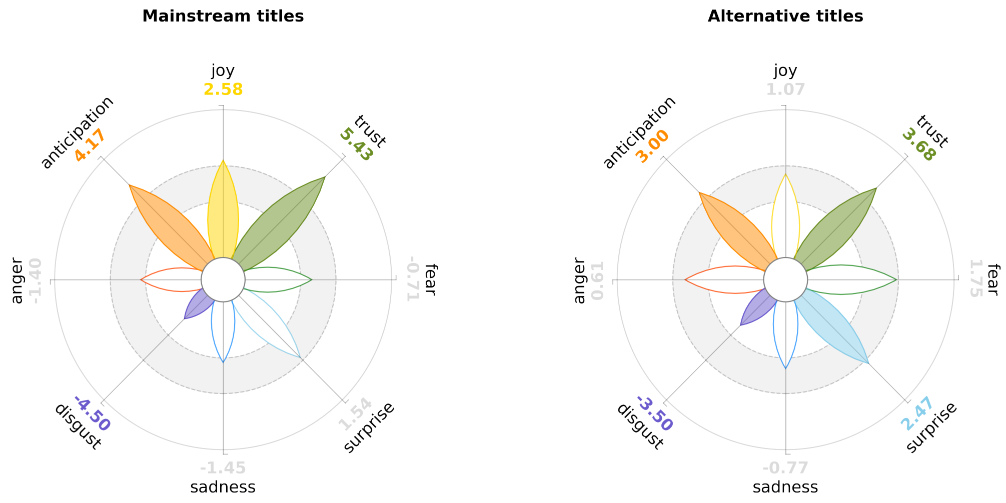

Figure 4. Emotions in mainstream and alternative titles. Emotions in mainstream titles resembles the ones in mainstream bodies, but with no surprise and low sadness. Alternative titles are again high in anticipation and trust, plus surprise, and low in disgust. Again, these distributions are significantly diverse from those in Fig. 1 (D).

regardless of the source of the title, thus producing a random selection of titles in both sub-lists. In no case the corresponding z-scores resembled those we found in the AstraZeneca's neighborhood: the only partition that produces such a Plutchik's flower is the one that follows the distinction between mainstream and alternative news. Notably, the low level of disgust seems to be consistent for general news and for our dataset, once reshuffled: in 68% of cases disgust was significantly lower than a neutral baseline.

References

1. Autorizzato il vaccino BioNTech/Pfizer. https://www.aifa.gov.it/-/autorizzato-il-vaccino-biontech-pfizer. [Online; accessed 7-December-2021].

2. AIFA: autorizzato vaccino AstraZeneca . https://www.aifa.gov.it/-/aifa-autorizzato-vaccino-astrazeneca. [Online; accessed 7-December-2021].

3. AIFA: sospensione precauzionale del vaccino AstraZeneca . https://www.aifa.gov.it/-/aifa-sospensione-precauzionale-del-vaccino-astrazeneca. [Online; accessed 7-December-2021].
